# Mechanism of MiR-145a-3p/Runx2 pathway in dexamethasone impairment of MC3T3-E1 osteogenic capacity in mice

**DOI:** 10.1371/journal.pone.0309951

**Published:** 2024-11-19

**Authors:** Hang Wu, Xinghua Liao, Tingrui Wu, Bin Xie, Sicheng Ding, Yiren Chen, Lijun Song, Bo Wei

**Affiliations:** 1 Orthopedics Center, Affiliated Hospital of Guangdong Medical University, Zhanjiang, China; 2 Central People’s Hospital of Zhanjiang, Affiliated Hospital of Guangdong Medical University, Zhanjiang, China; 3 Reproductive Medicine Center, Affiliated Hospital of Guangdong Medical University, Zhanjiang, China; University of Vermont, UNITED STATES OF AMERICA

## Abstract

**Objective:**

In this experiment, we screened key miRNAs involved in the dexamethasone-induced decrease in osteogenic capacity of mouse precursor osteoblasts MC3T3-E1 over and investigated their specific regulatory mechanisms.

**Methods:**

In this experiment, cell counting kit assay was utilized to act on MC3T3-E1 cells at 0, 5μM, 10μM, 15μM concentrations of dexamethasone for 24h, 48h and 72h to observe the changes in cell viability in order to select the appropriate dexamethasone concentration. Apoptosis and reactive oxygen species were detected by flow cytometry. The transcription of osteogenesis-related genes (Runx2, ALP, OCN, OPN, OPG, COL1A1) and protein expression levels (Runx2, ALP, OCN, OPN) were detected by Western Blot and qRT-PCR to validate the changes in cellular osteogenesis.

The differentially expressed miRNAs related to MC3T3-E1 osteogenic differentiation after dexamethasone action were screened out. The expression levels of selected target miRNAs were verified in the experimental group and the control group by qRT-PCR.

The miRNA inhibitor was transfected to knock down miRNA in dexamethasone-induced MC3T3-E1 injury. Alkaline phosphatase staining and flow cytometry were performed to detect apoptosis and reactive oxygen species changes. transcript and protein expression levels of osteogenesis-related genes in mouse MC3T3-E1 were detected by qRT-PCR and Western blot experiments.

By miRNA target gene prediction, luciferase reporter gene experiments, qRT-PCR and Western blot experiments were used to verify whether the selected target miRNAs targeted the target gene.

**Results:**

First, it was determined that 10μM dexamethasone solution was effective in inducing a decrease in osteogenic function in mouse MC3T3-E1 by CCK8 experiments, which showed a significant decrease in alkaline phosphatase activity, a decrease in calcium nodules as shown by alizarin red staining, an increase in apoptosis and reactive oxygen species as detected by flow cytometry, as well as a decrease in the expression of osteogenesis-related genes and proteins.

Five target miRNAs were identified: miR-706, miR-296-3p, miR-7011-5p, miR-145a-3p, and miR-149-3p. miR-145a-3p, which had the most pronounced and stable expression trend and was the most highly expressed miRNA, was chosen as the target of this experiment by qRT-PCR analysis. -145a-3p, as the subject of this experiment. Knockdown of miR-145a-3p in MC3T3-E1 cells after dexamethasone action significantly improved the expression of their impaired osteogenic indicators. It was shown that after knocking down the target miRNA, alkaline phosphatase staining was significantly increased compared with the dexamethasone-stimulated group and approached the level of the blank control group. Meanwhile, the expression of osteogenic function-related proteins and genes also increased in the dexamethasone-stimulated group after knocking down miR-145a-3p, and approached the level of the blank control group.

A direct targeting relationship between miR-145a-3p and Runx2 was indeed confirmed by luciferase reporter gene assays, qRT-PCR and Western blot experiments.

**Conclusions:**

The results indicated that dexamethasone impaired the osteogenic differentiation ability of MC3T3-E1 cells by inducing the up-regulation of miR-145a-3p expression. MiR-145a-3p inhibited the osteogenic differentiation ability of MC3T3-E1 cells by targeting and suppressing the expression level of Runx2 protein. Inhibition of miR-145a-3p levels significantly improved the osteogenic differentiation ability of MC3T3-E1 cells.

## Introduction

Osteoporosis, a global bone damage disease characterized by decreased bone mass and increased fragility, is a serious threat to people’s health. A variety of treatments for osteoporosis exist, including surgical and pharmacologic treatments, but the results are still unsatisfactory. Osteoblast dysfunction plays a key role in the progression of osteoporosis, so it is particularly important to find new strategies to modulate osteoblast function to improve osteoporosis. Glucocorticoid (GC), a widely used anti-inflammatory drug, plays an important role in the treatment of non-infectious inflammatory diseases such as rheumatoid arthritis [1], systemic lupus erythematosus [2], organ transplantation [3], asthma [4], and malignancies [5]. However, the use of glucocorticoids leads to osteoporosis [6] and fractures, making it the most common cause of drug-induced osteoporosis. This bone loss is particularly pronounced at trabecular-rich sites, leading to rapid bone loss, whereas at cortical bone-rich sites it shows a slow but continuous loss, putting patients at increased risk of fracture over time. Glucocorticoid-induced osteoporosis not only affects patients’ quality of life, but may also lead to high rates of disability and mortality [7], and has become an urgent public health problem worldwide. The pathogenesis of glucocorticoid-induced osteoporosis remains unclear, and it is generally recognized that impairment of osteoblast differentiation and bone formation is the main pathogenesis of glucocorticoid-induced osteoporosis (GIOP) [8,9]. Currently, clinically used antiresorptive drugs such as bisphosphonates [10] and calcitonin [11] have limited effect on improving the structure of bone trabeculae, although they can slow down the progression of the disease. Therefore, exploring the mechanism of decreased osteogenic function as well as searching for effective treatment remains a major task of research.

A growing body of research suggests that miRNAs play an important role in the onset and progression of a variety of diseases. Based on the important regulatory roles of miRNAs in skeletal physiology and pathology, and the potential link between hormonal bone diseases and specific miRNA expression changes. The following are some possible research avenues: identifying miRNAs related to hormonal bone disease: using high-throughput sequencing technology, comparing the expression profiles of miRNAs in patients with hormonal bone disease and healthy populations, and identifying miRNAs that are closely related to the occurrence and development of the disease, which may be directly or indirectly involved in the processes of bone metabolism, osteoblast proliferation and differentiation; exploring the effects of miRNAs on the Hormonal bone disease: through in vitro cellular experiments and in vivo animal models, to study how specific miRNAs regulate the proliferation, differentiation and apoptosis of osteoblasts, as well as the synthesis and degradation of bone matrix, and at the same time, to analyze how these miRNAs respond to hormonal stimulation, which in turn affects the state of bone health; to establish the causal relationship between miRNAs and hormonal bone disease: to utilize knockout, overexpression and other technology to verify the effect of specific miRNAs on hormonal bone disease in animal models, and to reveal the role of miRNAs in the pathogenesis of hormonal bone disease by comparing the skeletal phenotypes and biochemical indexes of experimental and control animals; develop miRNA-based therapeutic strategies for hormonal bone disease: based on the correlation between miRNAs and hormonal bone disease, we will research on the interventions targeting specific miRNAs, such as miRNA inhibitors and other therapeutic tools, such as the use of miRNA inhibitors. means, such as miRNA inhibitors or mimics, which are expected to provide new ideas and methods for the treatment of hormonal bone diseases by regulating the expression levels of these miRNAs [12]. At present, although studies have revealed the important roles of certain miRNAs in bone metabolic diseases, their specific mechanisms of action are still not clear enough. For example, some specific miRNAs can affect the biological properties of placental mesenchymal stem cells (MSCs) [13], such as the abnormal elevation of miR-705, which binds to its target genes and alters the biological properties of MSCs, inhibiting their osteogenic differentiation and increasing their lipogenic capacity [14]. On the contrary, inhibition of miR-705 expression increases the levels of proteins translated after transcription of homology box gene A10 (HomeoboxA10, HOXA10) and forkhead box transcription factor subfamily O (Forkhead box gene group O, FoxO1), which is a key component of bone marrow mesenchymal stem cells (MSCs) to inhibit the osteogenic differentiation and lipogenic differentiation of MSCs by promoting the formation of calcified nodules. lipidogenic differentiation, thereby delaying the onset of osteoporosis [15]. Previous studies have identified a new miRNA, miR-2861, which is closely related to osteoporosis. miRNA target gene is Histone deacetylase 5 (HDAC5), which upregulates Runx2 expression and promotes osteoblastic differentiation [16,17]. When miR-2861 was knocked down in mice, osteoblast differentiation was blocked and bone density decreased. Meanwhile, mutations in the miR-2861 precursor locus are closely related to the development of osteoporosis in humans. In addition to miR-2861, several other miRNAs have been found to have an effect on lipogenesis and osteogenic differentiation. For example, miR-152 belongs to the miR-148 family and plays an important role in tumors, while its specific mechanism of action in osteoporosis is yet to be further investigated [18]. In addition to the above mentioned microRNAs, there are some other miRNAs that also have an impact on the process of lipogenic and osteogenic differentiation. For example, the expression level of miR-335 is relatively high in human mesenchymal stem cells (hMSCs) in the resting state, however, its expression gradually decreases as the osteogenic process proceeds [19]. However, when miR-335 is overexpressed, it exerts an inhibitory effect on the osteogenic and lipogenic differentiation ability of hMSCs. Further studies revealed the underlying mechanism: miR-335 regulates the differentiation process of hMSCs by directly targeting Runx2, a key factor [20]. It has also been noted that miR-138 has a role in inhibiting the lipogenic and osteogenic differentiation of MSCs [21]. In contrast, miR-20a promotes adipogenesis by acting on transforming growth factor β2 (TGFBR2) [22] and lysine-specific histone demethylase 6B (KDM6B) [23]. Notably, in adipose stem cells (ADSCs), miR-26a inhibits osteogenic differentiation by acting on Smad homolog 1 (Smad1) [24], whereas in bone marrow mesenchymal stem cells (BMSCs), it inhibits osteogenic differentiation through the regulation of serine/threonine kinase (GSK3β) [25] and Tob1 [26]. miR-30e is also quite distinctive in that when it targets low-density lipoprotein receptor-related protein 6 (LRP6) [27], it can simultaneously promote lipogenesis and inhibit osteogenesis, whereas when it targets insulin-like growth factor 2 (IGF2) [28], it mainly inhibits osteogenesis. Another study indicated that miR-153 inhibits osteogenesis by targeting bone molding protein receptor 2 (BMPR2) [29]. Not only that, but a variety of miRNAs have been found to play important roles in lipogenesis and osteogenic differentiation. For example, miR-188 inhibits osteogenic function by targeting HDAC9 [30] and RICTOR [31], as well as miR-223 by targeting fibroblast growth factor receptor 2 (FGFR2) [32] and miR-320 by targeting Runx2 [33], which all promote lipogenic differentiation while inhibiting osteogenic function. However, miR-194 has a slightly different mechanism of action, which promotes osteogenic differentiation and lipogenic differentiation by targeting chicken ovalbumin upstream promoter transcription factor II (COUP-TFII) [34]. Furthermore, it has been shown that miR-455-3p promotes chondrogenesis by targeting Runx2 [35]. In summary, the study of miRNAs in association with hormonal bone disease can help to deeply understand the pathogenesis of hormonal bone disease and provide new strategies for disease prevention and treatment. However, research in this field is still in its infancy and needs to be supported by more experimental evidence and clinical data.

Runx2, a member of the RUNX family, is centrally characterized by the possession of the DNA-binding structural domain Runt. this family consists of three members, Runx1, Runx2, and Runx3, of which Runx2 significantly enhances its DNA-binding ability and protein stability by heterodimerization with Cbfb [36]. runx2 possesses two promoters, P1 and P2, which are responsible for encoding different types of Runx2 proteins—transcripts from P1 encode type II Runx2, while transcripts from P2 encode type I Runx2 [37]. In terms of cellular expression, Runx2 functions mainly in osteoblasts and chondrocytes [38]. Particularly during the development of chondrocytes, Runx2 expression shows a clear phase change: during the quiescent phase, its expression is weak; after entering the pre-hypertrophic phase, the expression is up-regulated and persists until the late hypertrophic chondrocytes. Notably, when the Runx2 gene is defective (e.g., Runx2-/- mice), osteoblasts and bone formation are severely affected and the maturation process of chondrocytes is significantly inhibited [39]. Col2a1 expressed in immature chondrocytes is expressed throughout Runx2 gene-deficient bone [40], whereas Col1a1 expressed in hypertrophied chondrocytes is restricted to tibia, fibula, radius, and ulna [41]. Runx2 is functionally diverse and critical, with an important function being in osteogenesis through osteoblast-specific cis-acting elements (OSEs) and trans-activation of major bone matrix protein genes during bone formation [42]. In addition, Runx2 acts as a transcription factor together with core binding factor β (Cbfβ) [43], forming a heterodimer that regulates the expression of osteoblast-associated genes including Col1a1, Spp1, BGLAP/OCN and IBSP [44,45]. The expression level of Runx2 also varies at different developmental stages of osteoblasts. Runx2 is expressed in unlocalized mesenchymal cells; its expression level is up-regulated in precursor cells of osteogenesis; it reaches a maximum in immature osteoblasts; and it is down-regulated in mature osteoblasts [46,47]. This expression pattern suggests that Runx2 not only regulates the proliferation of osteogenic precursor cells, but also participates in the regulation of bone matrix protein gene expression [48]. In addition, Runx2 plays an important role in the differentiation of bone marrow mesenchymal cells to the osteogenic lineage [49] and is required for the growth of whole-bone precursor osteoblasts and suture mesenchymal cells. Therefore, it can be hypothesized that Runx2 may contribute to the differentiation of bone marrow mesenchymal or osteogenic precursor cells to the osteoblast lineage by promoting their proliferation and, consequently, their differentiation to the osteoblast lineage.Finally, the functional realization of Runx2 is not isolated; it co-regulates the proliferation and differentiation of osteoblasts through interactions with major signaling pathways, such as Fgf, hedgehog, Wnt, and Pthlh [50], as well as major transcription factors, such as Sp7 [51], Dlx5, and Mef2 [52]. This process reflects a complex and fine regulatory network within the cell, in which the mutual regulation among transcription factors may be one of the key mechanisms to achieve osteoblast proliferation and differentiation [53].

## Materials and methods

### Cell culture and osteogenic induction

MC3T3-E1 mouse osteogenic precursor cells were cultured in MEM-Alpha (α-MEM) at 37°C with 5% CO2 and 10% FBS and 1% penicillin-streptomycin. As described in prior research, MC3T3-E1 cells were cultured in osteogenic differentiation medium containing 10% fetal bovine serum, α-MEM, and 1% penicillin-streptomycin. The medium also contained 100 nM DEX, 50 μM ascorbic acid, and 10 mM β-phosphoglycerol. The cells were in fusion at 70%.

### CCK8 assays

MC3T3-E1 cells in the logarithmic growth phase were obtained and inoculated into 96-well plates containing 2.5×103 cells per well and approximately 100μl of complete culture medium. The cells were subsequently subjected to treatment with dexamethasone at varying concentrations (2μM, 6μM, 18μM) for time intervals of 24 hours, 48 hours, 72 hours, and 96 hours. The absorbance was measured at 450 nm using a full-wavelength enzyme marker and the cell counting kit (CCK-8) reagent (cat. no. C0039; Beijing Solarbio Science and Technology Co. Ltd.) to determine the viability of cells. By constructing a graph using the OD value of each well, it was possible to deduce the trend of the corresponding cell viability. In conclusion, the DEX concentration required for subsequent experiments was ascertained.

### Analysis of apoptosis

The quantification of apoptosis percentage in MC3T3-E1 cells was performed utilizing the Annexin V-FITC Apoptosis Detection Reagent (Beyotime Biotechnology, catalog number C1062). Following treatment of cells with 10μM DEX, they were resuspended in 5μL Annexin V-FITC and 10μL propidium iodide staining solution, respectively. Following this, the cells were incubated at room temperature for 30 minutes in a flow-through tube. Ultimately, the outcomes of the examination were identified through the utilization of flow cytometry (BD Biosciences, Franklin Lakes, NJ, USA).

### Analysis of reactive oxygen species (ROS)

For the detection of DCFH-DA, the DCFH-DA fluorescent probe detection reagent (cat. no. D6470; Beijing Solarbio Technology Co. Ltd.) was utilized. To achieve a final concentration of 10 μmol/L, the DCFH-DA was diluted with serum-free culture medium in a 1:1000 ratio. The cells were gathered and agitated in the diluted DCFH-DA at a concentration ranging from one million to twenty million cells/mL. Approximately one milliliter of DCFH-DA was used per well to contain the cells. For each cell volume per well, an approximate volume of 1 ml of diluted DCFH-DA was utilized. Subsequently, the wells were incubated in a light-protected cell culture incubator set at 37°C with 5% CO2. Following this, the cells were incubated for 20 minutes at 37°C, 5% CO2, in a light-protected cell culture incubator. During this time, the cells were inverted and mixed every 3 to 5 minutes to ensure that the probe made complete contact with the cells. To eliminate any DCFH-DA that failed to enter the cells, they underwent a minimum of three washes with serum-free cell culture medium. Following this, the precipitate was reconstituted with a suitable quantity of serum-free medium and transferred to flow tubes. Ultimately, the findings were assessed via flow cytometry (BD Biosciences, Franklin Lakes, NJ, USA).

### Alkaline phosphatase (ALP) staining

Following the induction of osteogenic differentiation in MC3T3-E1 cells, the osteogenic differentiation medium was discarded and the cells underwent three washes with 1ΗPBS. The cells were fixed in 4% paraformaldehyde at 25˚C for a duration of 30 minutes. Subsequently, they were washed 3–5 times with washing solution for an additional 3–5 minutes each time. Following the removal of the washing solution, a suitable quantity of staining working solution was added to envelop the cells. The cells were incubated at room temperature and shielded from light until the desired shade was achieved. Once the staining working solution was removed, the color reaction was terminated by washing the cells with distillate. The Leagene BCIP/NBT Alkaline Phosphatase Chromogenic Kit (cat. no. PW0078; Beijing Leagene Biotechnology Co. Ltd.) was utilized to conduct the alkaline phosphatase staining.

### Alizarin red (ARS) staining

Following the induction of osteogenic differentiation in MC3T3-E1 cells, the osteogenic differentiation medium was extracted and the cells were rinsed three times with 1ΗPBS. The cells were fixed for 30 minutes at room temperature with 4% paraformaldehyde. Subsequently, they were washed 3–5 times with 1–3 minutes of 1ΗPBS. Following the removal of the washing solution, 2 ml of alizarin red working solution was added to each well, and the cells were stained for 5–10 minutes at room temperature. Any remaining staining working solution was removed by rinsing with 1×PBS. Following the addition of 2 ml of 1ΗPBS to each well, images were captured of the calcified nodules using an inverted light microscope operating at a magnification of X100. Utilizing OriCell Alizarin Red Staining Solution (cat. no. ALIR-10001; Guangzhou OriCell Biotechnology Co. Ltd.), alizarin red staining was accomplished.

### Screening and determination of target miRNAs

A small subset of miRNAs that exhibited unique correspondences with the osteogenesis-related genes Runx2, ALP, OCN, and OPN as predicted on the website was obtained by mapping the intersection of these four distinct miRNAs. Prior to further analysis, the following miRNAs were eliminated from consideration: miR-706, miR-296-3p, miR-7011-5p, miR-145a-3p, and miR-149-3p. 7011-5p, miR-145a-3p, and miR-149-3p, and the primer sequences were synthesized as described below (Table 1). Following the detection of each miRNA with qRT-PCR, one miRNA that exhibited stable expression throughout the process of osteogenesis was selected for utilization in the subsequent experiments.

**Table 1:** MiRNA primer sequences.

Name	Sequences (5′→ 3′)
miR-145a-3p	Forward: GTCCAGTTTTCCCAGGAATCCCT Reverse: ATTCCTGGAAATACTGTTCTTG
miR-706	Forward: AGAGAAACCCUGUCUCAAAAAA Reverse: UGAGACAGGGUUUCUCUUUUU
miR-296-3p	Forward: TGGGAGGGCCCCCCCTCAA Reverse: TGGTGTCGTGGAGTCG
miR-7011-5p	Forward: AGGAGGAUGGGAGAGGGAGGUGU Reverse: UCUGCUUCCCUCCUCCUCUAG
miR-149-3p	Forward: TCTGGCTCCGTGTCTTCACTCCC Reverse: GAGGGAGGGACGGGGGCGGTGC
U6	Forward: TCCGATCGTGAAGCGTTC Reverse: GTGCAAGGGTCCGAGGT

### Cell transfection

Suzhou Jimma Genetics Co. Ltd. produced miR-145a-3p mimic (5’-AUUUCCUGGAAAUACUGUUCUUG-3’), miR-145a-3p inhibitor (5’-CAAGAACAGUAUUUUCCAGGAAU-3’), its corresponding negative control (5’-UUUUUCCGAACGUGUCACGUTT-3’), and NC-inhibitor (5’-CAGUACUUUUGUGUAGUACAA-3’). In response to changes in miR-145a-3p expression or downregulation, these compounds were synthesised. Six-well plates were inoculated with 2 ml of cell suspension and incubated at 37°C with 5% CO2 for one night. At the 60%-70% fusion stage, MC3T3E1 cells were transfected with Lipofetamine 3000 and 5 μL of inhibitor or mimetic. Following a period of 4–6 hours at 37˚C, complete culture medium (α-MEM containing 10% fetal bovine serum) was substituted for serum-free transfer medium. The incubation period was extended to 48 hours. The efficacy of the transfection was assessed by comparing the levels of miR-145a-3p expression in the untransfected and transfected samples.

### Reverse transcription-quantitative chain reaction for polymerase

RNA was isolated from MC3T3-E1 cells utilizing RNA-easy Isolation Reagent (Nanyang Novozymes Bioscience Co., Ltd., catalog number R701). The PrimeScript RT reagent Kit with gDNA Eraser kit (cat. no. RR047A; Beijing Baozhi Doctor Bio-Technology Co., Ltd.) was utilized to extract RNA samples from MC3T3-E1 cells. Subsequently, the TB Green™ Premix Ex Taq™ II (Tli RNaseH Plus) (cat. no. RR820A; Beijing Baori Physics Technology Co. The thermal cycling conditions for the quantitative polymerase chain reaction were as follows: 95°C for 30 seconds; 40 cycles of 95°C for 5 seconds and 60°C for 34 seconds; and final extensions of 95°C for 15 seconds, 60°C for 1 minute, and 95°C for 15 seconds. The calculations for the relative levels of Runx2 and miR-145a-3p mRNA utilized U6 and GAPDH as internal controls, respectively. The sequences of the primers were as follows (Table 2).

**Table 2:** Osteogenesis-related primer sequences.

Name	Sequences (5′→ 3′)
Runx2	Forward: TCCAGACCAGCAGCACTCCATATC Reverse: CTTCCGTCAGCGTCAACACCATC
ALP	Forward: AGTCAGCTGAAGTCTGGGAG Reverse: CTGCTTCCGAGACAGAGAGG
OCN	Forward: CAAGCAGGAGGGCAATAAGGTAGTG Reverse: TGCGTTTGTAGGCGGTCTTCAAG
OPN	Forward: ATGGACGACGATGATGACGATGATG Reverse: CTTGTGTACTAGCAGTGACGGTCTC
COL1A1	Forward: TCTGCGACAACGGCAAGGTG Reverse: GACGCCGGTGGTTTCTTGGT
OPG	Forward: CCATCGGGTTCCCATAAAGTCAGT Reverse: AAAGCCCCAAAGTAGTACGTCGCATCT
GAPDH	Forward: GGCAAATTCAACGGCACAGTCAAG Reverse: TCGCTCCTGGAAGATGGTGATGG

### Western blot analysis

The protein concentration was determined using the BCA Protein Concentration Assay Kit (Enhanced) (cat. no. P0010; Beyotime Biotechnology) after the target proteins were extracted from MC3T3-E1 cells using RIPA lysate (strong) (cat. no. P0013B; Beyotime Biotechnology). Proteins were transferred to a PVDF membrane following separation by 10% sodium dodecyl sulfate-polyacrylamide gel electrophoresis. After sealing the membranes with 5% skimmed milk at 4 degrees Celsius for one hour, they were incubated with the subsequent primary antibodies: anti-Runx2, OCN, ALP, and GAPDH. while shaken at 4 degrees Celsius overnight. Following the initial incubation, the membranes were subjected to an additional incubation using goat anti-rabbit IgG (H+L) labeled with horseradish peroxidase (1:5000, cat. no. A0208, Biyoung Biotech). One hour of incubation was conducted at room temperature on an agitator. The quantification of the signal was performed using a Tanon5200 chemiluminescence imager, while the densitometry of the bands was performed using ImageJ software (version 1.53; National Institutes of Health, USA).

### Dual luciferase reporter assay

The binding site of runx2 and miR-145a-3p was determined by employing the bioinformatics application TargetScan 7.2 (Targetscan.org/vert_72/) to predict the target gene of miR-145a-3p. Following this, we constructed a reporter vector for the target gene by inserting the cloned plasmid into a dual-luciferase reporter vector—which is capable of measuring fluorescence intensity—and comprises luciferase and a fluorescent substrate for the luciferase reaction—and intercepted the plasmid within the 500-2000bp length region based on the binding site. To express the target gene, the constructed reporter vector is transfected into target cells using Lipofetamine 3000. To facilitate fluorescence in response to luciferase, the luciferase substrate is added to the cell culture solution. The evaluation of the target gene’s expression level can be accomplished through the utilization of a fluorescence analyzer to quantify the intensity of fluorescence.

### Statistical analysis

For the quantitative analysis, GraphPad Prism 8.0 was utilized. Each experiment was independently replicated three times. The statistical measures are mean and standard deviation. The unpaired t-test, two-way ANOVA with Bonferroni’s multiple comparison test, or one-way ANOVA with Tukey’s multiple comparison test were employed to examine the differences between the groups. Differences were deemed statistically significant when *P<0.05, **P<0.01, ***P<0.001,****P < 0.0001.

### Statement of ethics

The PubChem, PharmMapper, Swiss Target Prediction, Uniprot, OMIM, TTD, Genecards, and STRING databases are publicly available and allows researchers to download and analyze. qqqqethical approval for open public databases that we used in this work.

## Results

### Cell viability assay for mouse MC3T3-E1

**Fig 1:**
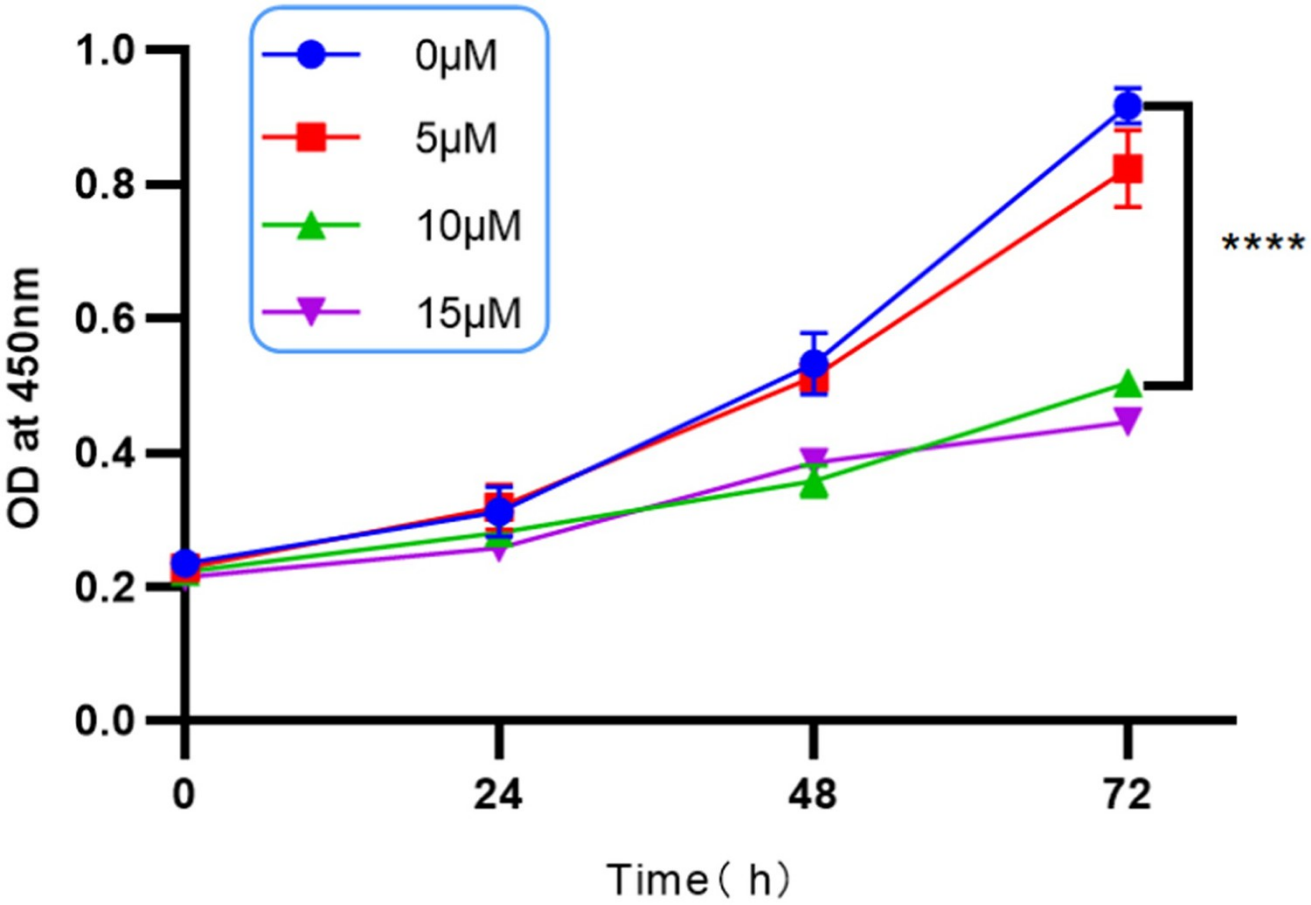
Cell viability detected by CCK-8 cell proliferation assay. CCK8 test results represent absorbance at different concentrations at different times and were most significant at 10μM DEX and 72h; * represents statistical differences between treatment groups, ****P < 0.0001.

To assess the effect of dexamethasone solution on the viability of MC3T3-E1 cells, mouse MC3T3-E1 cells were cultured with 5μM, 10μM and 15μM dexamethasone, and the absorbance of the treated cells at 450nm was determined by CCK-8 cell proliferation assay at 24h, 48h and 72h, respectively. The results of the experiment showed that at 72h, the difference in cell viability between the blank group and the group using 5μM DEX was not significant, while the value of cell viability between the 10μM and 15μM groups was not statistically significant (P = 0.4814) (Fig 1), so the effect of the present experiment on the viability of MC3T3-E1 cells was most significant when 10μM DEX was used, and therefore, an experimental concentration of 10μM for dexamethasone solution was selected for the subsequent experiments. μM was used as a follow-up experiment.

### Effect of dexamethasone stimulation at 10 μM concentration on apoptosis and reactive oxygen species in MC3T3-E1 cells

**Fig 2:**
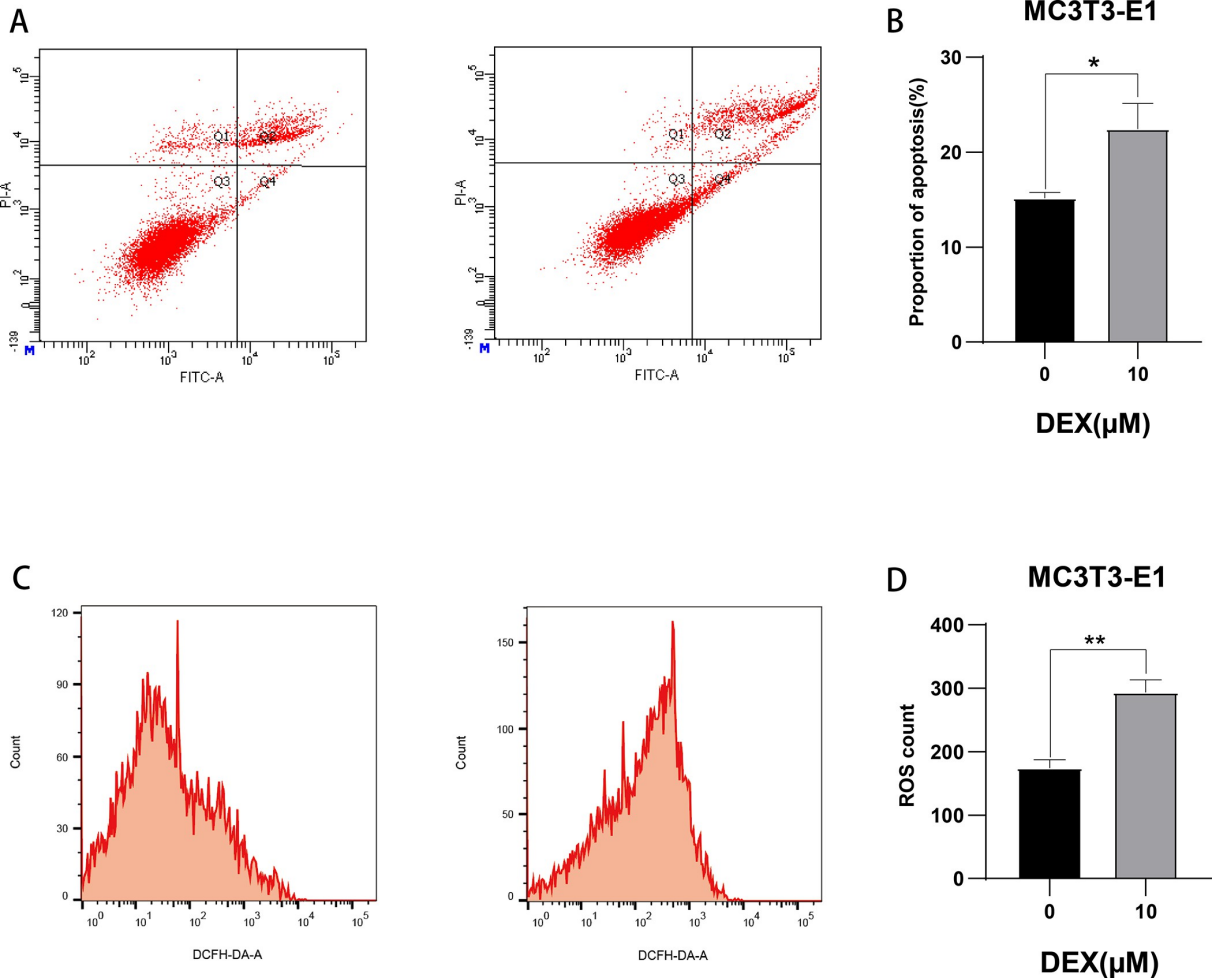
Flow cytometry detection of apoptosis and ROS. A: the second and fourth quadrants in the flow cytogram indicate early apoptosis and late apoptosis, respectively; B: bar graph statistics of apoptosis proportion; C; the value of the highest peak corresponding to the horizontal coordinate in the flow cytogram indicates the magnitude of reactive oxygen species; D: bar graph statistics of changes in reactive oxygen species; *represents the statistical differences between different treatment groups, *P < 0.05, **P < 0.01.

After incubating mouse MC3T3-E1 cells with 10μM dexamethasone for 72h (control group without dexamethasone), the change of apoptosis rate was analyzed quantitatively by flow cytometry through Annexin V-FITC/PI double staining method, and the experimental results showed that the apoptosis rate of MC3T3-E1 cells reached 22.5±5.1(%) after 10μM dexamethasone stimulation for 72h (Fig 2A and 2B), indicating that dexamethasone stimulation at 10μM concentration promotes osteoblast apoptosis; and then the same operation was followed by flow cytometry to quantitatively analyze the alteration of reactive oxygen species (ROS), and the experimental results showed that after 10μM dexamethasone stimulation for 72h, the reactive oxygen species in the MC3T3-E1 cells increased by 1.3±0.1 times (Fig 2C and 2D), indicating that the 10μM concentration of dexamethasone stimulation promotes an increase in osteoblast reactive oxygen species.

### Effect of dexamethasone stimulation at 10μM concentration on alkaline phosphatase and mineralized nodules

**Fig 3:**
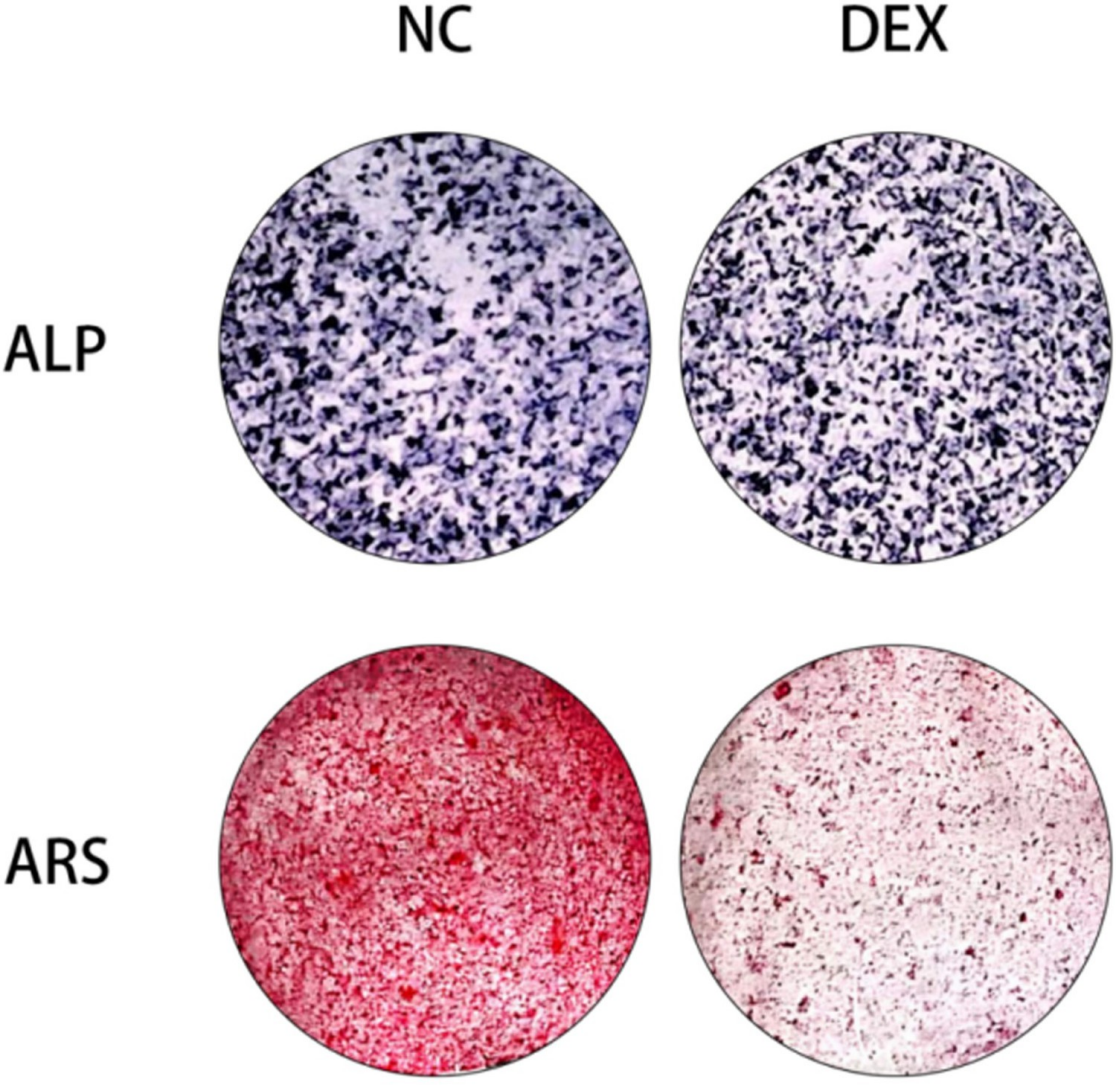
Alkaline phosphatase and alizarin Red staining. In gross observations of alkaline phosphatase staining and alizarin red staining, respectively, both significant reductions in alkaline phosphatase content and mineralized nodules were observed upon stimulation of preosteoblast cells with DEX at a concentration of 10 μM.

In order to assess the effect of MC3T3-E1 cells stimulated by dexamethasone solution on alkaline phosphatase production, osteogenic induction with 100nM DEX, 50μM ascorbic acid and 10mM β-phosphoglycerol was carried out firstly for 5 days, followed by incubation of mouse MC3T3-E1 cells with 10μM dexamethasone for 72h (control without dexamethasone), and then an alkaline phosphatase staining kit was used to detect the alkaline phosphatase content, and the experimental results illustrated that after osteogenic induction reagent culture and 10μM dexamethasone stimulation, the alkaline phosphatase content in MC3T3-E1 cells was reduced (Fig 3 ALP), indicating that dexamethasone causes a reduction in alkaline phosphatase content in MC3T3-E1 cells; also, in order to assess the effect of stimulation of MC3T3-E1 cells by dexamethasone solution on alkaline phosphatase production, the MC3T3-E1 cells were stimulated with 100 nM DEX, 50 μM ascorbic acid and 10 mM β-phosphoglycerol with the help of a β-phosphatidylglycerol kit. Osteogenic induction was first performed for 15 days, followed by incubation of mouse MC3T3-E1 cells with 10μM dexamethasone for 72h (control without dexamethasone), and the mineralized nodule content was detected by using an alizarin red staining kit.The results of the experiment illustrated that after incubation with osteogenic induction reagents and 10μM dexamethasone caused a decrease in mineralized nodules within MC3T3-E1 cells (Fig 3 ARS).

### Effect of dexamethasone stimulation at 10μM concentration on the expression of osteogenesis-related proteins

**Fig 4:**
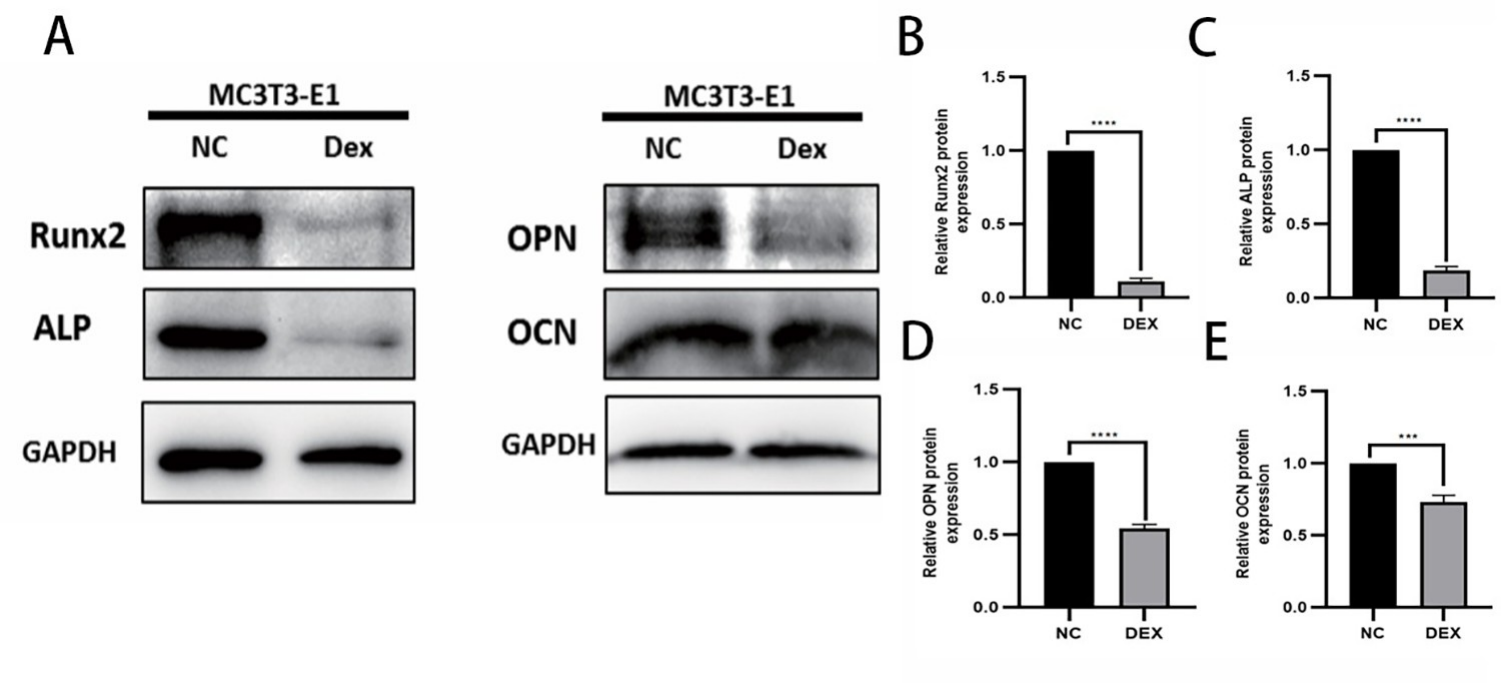
WB sensing osteogenesis-related protein expression. A: total protein bands; B-E: the expression of Runx2, ALP, OPN, and OCN, respectively, indicated by the statistical analysis of the gray value to make a histogram according to the protein bands shown; * represents the statistical difference between different treatment groups, ***P < 0.001, ****P < 0.0001.

In order to assess the effect of dexamethasone solution on the expression of osteogenic proteins in MC3T3-E1 cells, mouse MC3T3-E1 cells were incubated with 10μM dexamethasone for 72h (control without dexamethasone), the expression trend of osteogenic related proteins Runx2, ALP, OCN and OPN was analyzed by Western blot experiment and Tanon 5200 exposure strip statistics. The experimental results showed that after stimulating osteoblasts with 10μM dexamethasone for 72h, the osteogenic related proteins showed a significant down-regulation trend (Fig 4), indicating that the 10μM concentration of dexamethasone inhibited the osteogenic function to a certain extent.

### Effects of dexamethasone stimulation at 10μM concentration on the expression of osteogenesis-related genes

**Fig 5:**
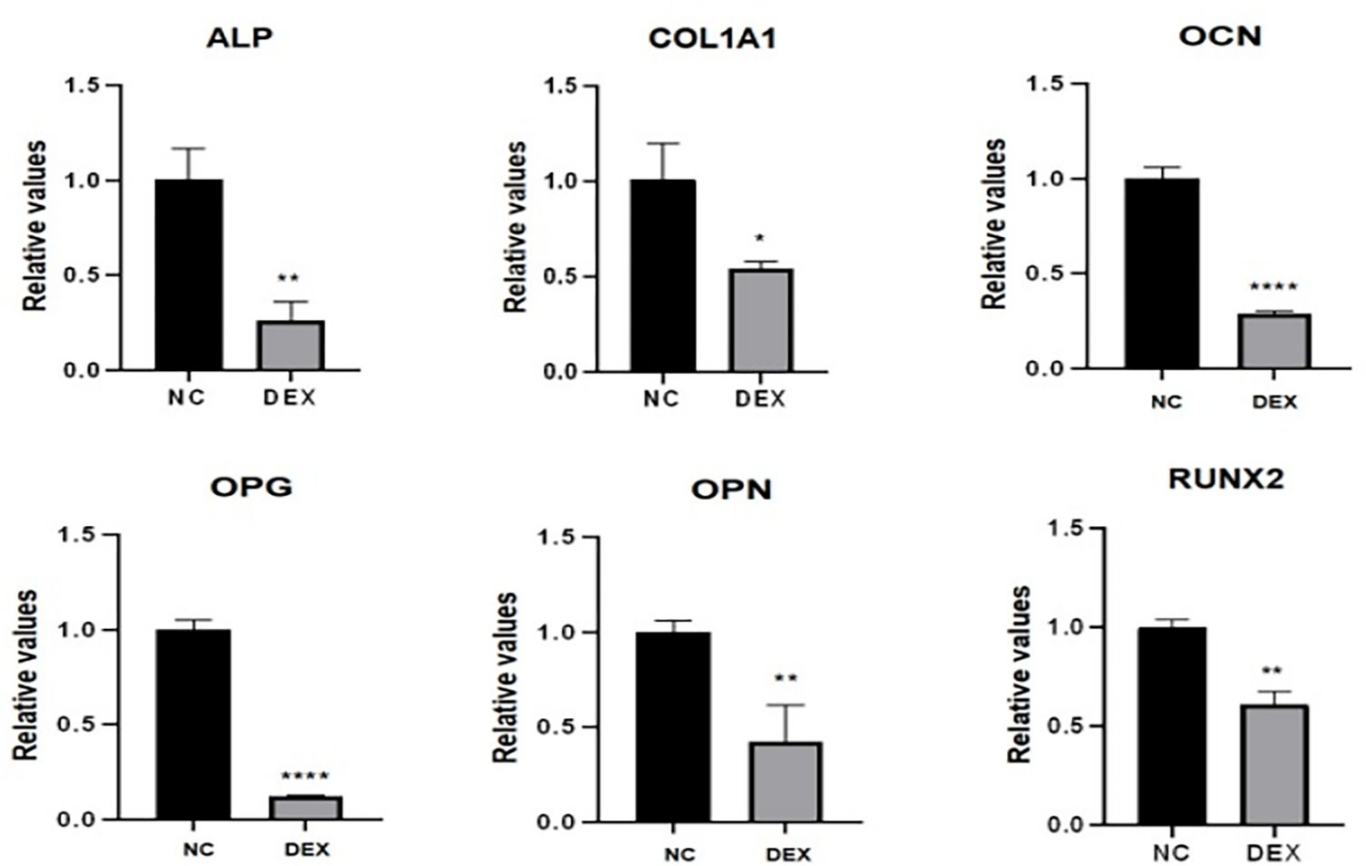
PCR sensing the expression of osteogenesis-related genes. The expression of ALP, COL1A1, OCN, OPG, OPN, and Runx2 genes was detected by PCR, and histogram analysis was done based on the CT values, which showed that all of these genes showed a trend of low expression; * represents the statistical differences between different treatment groups, *P < 0.05, **P < 0.01, ****P < 0.0001.

To assess the effect of dexamethasone solution on osteogenic gene expression in MC3T3-E1 cells, after incubating mouse MC3T3-E1 cells with 10μM dexamethasone for 72h (control without dexamethasone), the expression of osteogenesis-related genes Runx2, ALP, OCN, OPN, OPG, and COL1A1 was analyzed statistically by qRT-PCR experiments, ABI7500 to Trend. The experimental results showed that after stimulating osteoblasts with 10μM dexamethasone for 72h, the expression of osteogenic-related genes were all significantly down-regulated compared with the NC group (Fig 5), indicating that 10μM dexamethasone inhibited osteogenic gene expression to a certain extent.

### Targeted screening of miRNAs and validation

**Fig 6:**
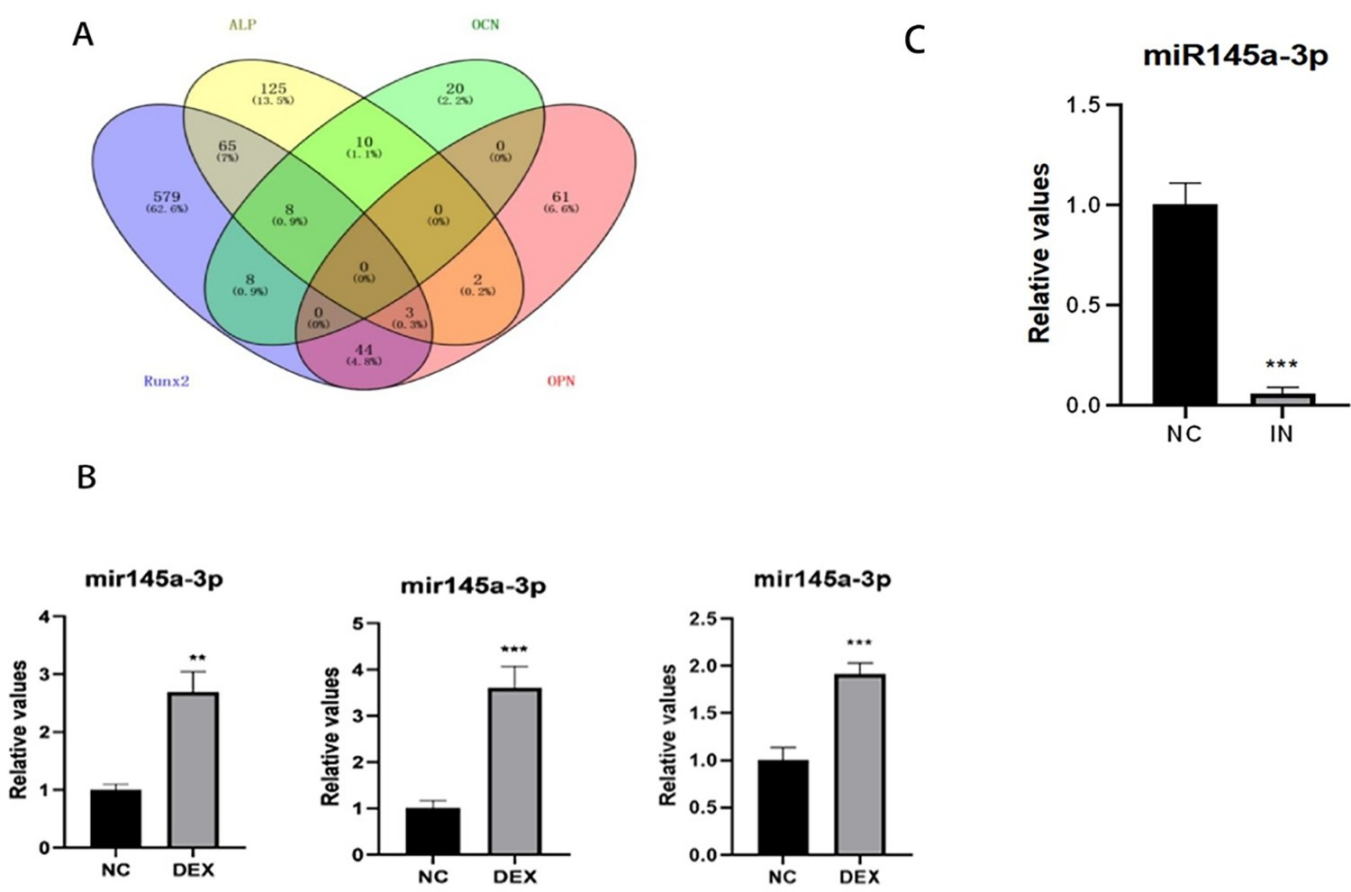
Target miRNAs screening. A: the screened target miRNAs of each category were compared to take the intersection; B: PCR was used to verify the expression trend of the screened miRNAs respectively; C: the transfection effect was verified by PCR after knocking down miR-145a-3p by using the appropriate conditions; * represents the statistical differences of different treatment groups, **P<*0*.01, ***P<*0*.001.

Firstly, four groups of miRNAs were screened according to the targeting of osteogenic genes Runx2, ALP, OCN and OPN, and then five miRNAs most likely to be involved in the regulation of osteogenesis were obtained by comparing with each other and taking the intersection of the five miRNAs (Fig 6A): miR-706, miR-296-3p, miR-7011-5p, miR-145a-3p and miR-149-3p. 149-3p; the only stable and highly expressed miR-145a-3p was further verified and confirmed by qRT-PCR to be used as the target miRNA for the subsequent experiments (Fig 6B); miR-145a-3p was subsequently knocked down in the state of decreased osteogenic capacity by cell transfection experiments, and then the expression of the knocked down miRNAs was investigated by qRT-PCR, and the test results showed that the six-wells could be used as a target for the subsequent experiments, and that the six-wells could be used as a target for the subsequent experiments. The results showed that miR-145a-3p could be successfully knocked down by adding 5 μl miR-145a-3p Inhibitor (pre-dissolved and configured according to the instructions), 3.75 μl Lipo3000, and 250 μl OPTI-MEM to each well of a six-well plate (Fig 6C).

### Effect of knockdown of miR-145a-3p on apoptosis and reactive oxygen species in MC3T3-E1

**Fig 7:**
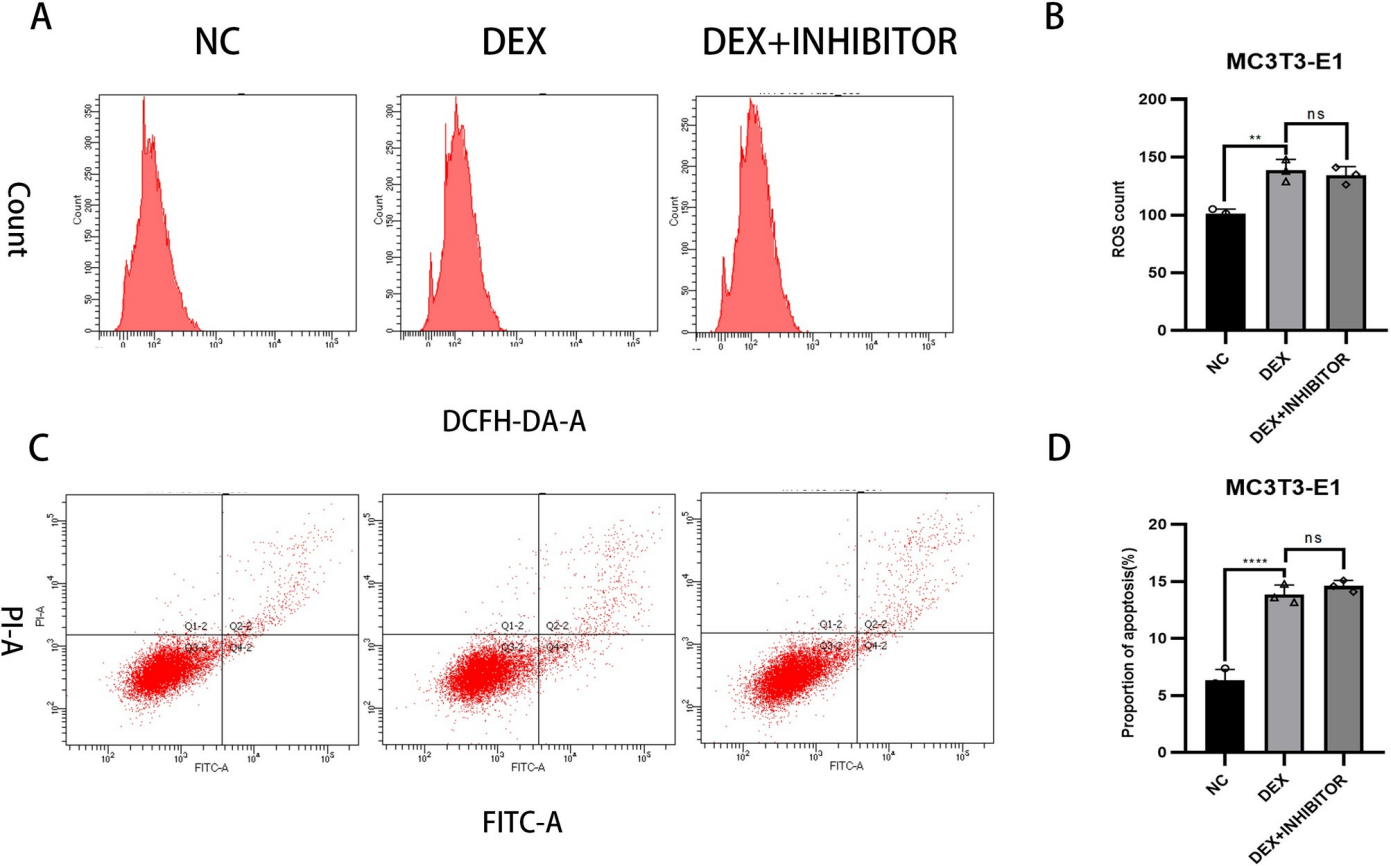
Flow cytometry detection of apoptosis and reactive oxygen species changes. A: flow cytometry detection of reactive oxygen species; B: quantitative analysis of reactive oxygen species; C: flow cytometry detection of apoptosis: D: quantitative analysis of apoptosis rate; * represents the statistical difference between the different treatment groups, **P < 0.01, ****P < 0.0001.

This experiment was divided into three groups, the NC group was not treated, the DEX group was added with 10μM dexamethasone, and the last group was added with both 10μM dexamethasone and transfection to knock down miR-145a-3p; firstly, the changes in reactive oxygen species were quantitatively analyzed by flow cytometry, and the results of the experiments showed that the changes in reactive oxygen species were similar to that of the DEX group after knocking down miR-145a-3p (Fig 7A and 7B) Subsequently, the changes in apoptosis rate were quantified by flow cytometry through Annexin V-FITC/PI double staining, and the experimental results showed that the changes in apoptosis rate after knocking down miR-145a-3p were close to that of the DEX group (Fig 7C and 7D); the above results indicated that miR-145a-3p did not act by affecting apoptosis and r. eactive oxygen species.

### Effects on alkaline phosphatase after knockdown of miR-145a-3p

**Fig 8:**
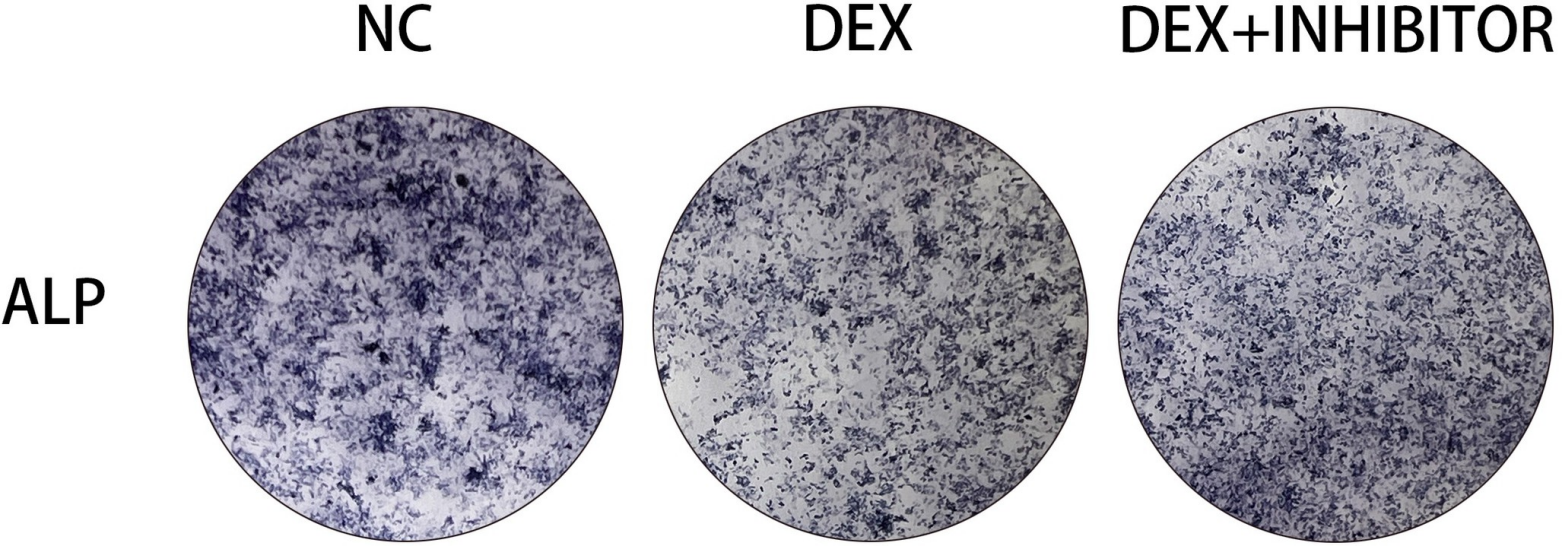
Alkaline phosphatase staining. Observation of alkaline phosphatase staining showed an increase in the originally reduced alkaline phosphatase content after knocking down miR-145a-3p.

In order to investigate whether the already reduced alkaline phosphatase content would increase after knocking down miR-145a-3p, the experiment was divided into three groups, and the alkaline phosphatase content was detected using alkaline phosphatase staining kit after knocking down using the transfection method. The results of the experiment showed that the alkaline phosphatase content, which would have been reduced because of dexamethasone stimulation, would rise after knocking down miR-145a-3p (Fig 8), indicating that miR-145a-3p could ameliorate the reduction in alkaline phosphatase content due to dexamethasone stimulation.

### Effect of knockdown of miR-145a-3p on the expression of osteogenesis-related proteins

**Fig 9:**
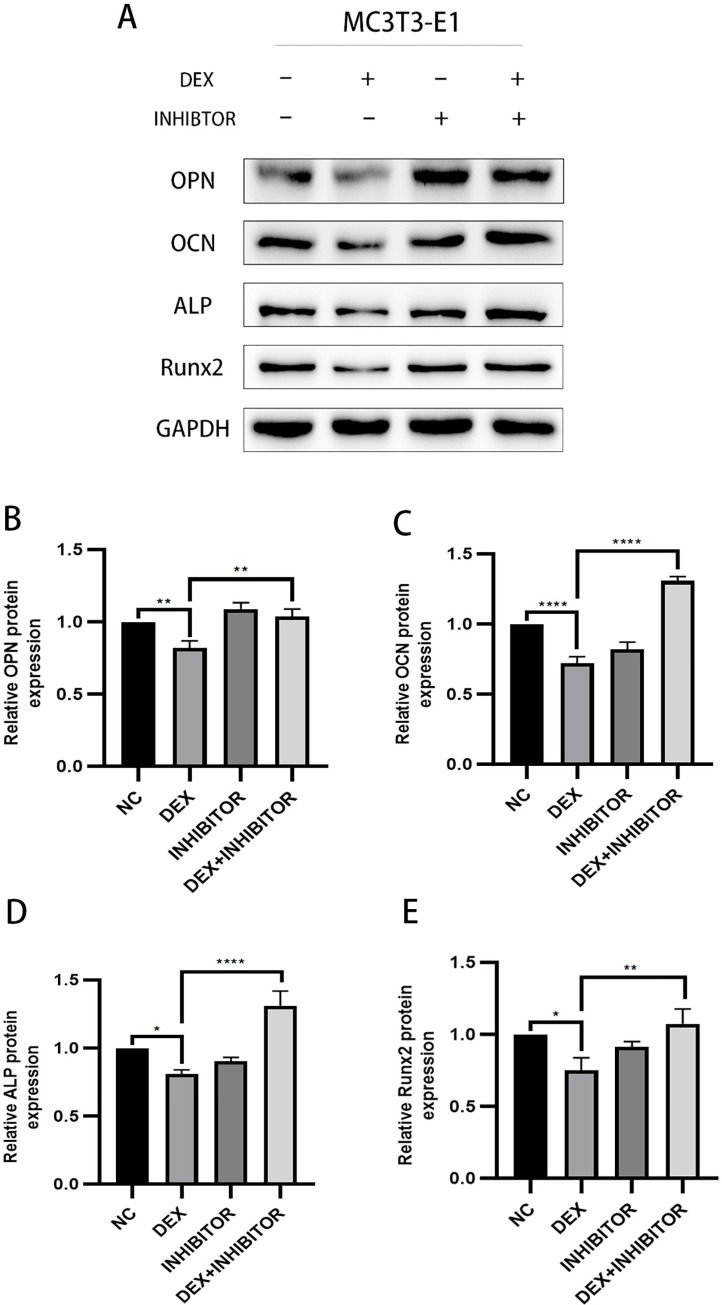
WB sensing osteogenesis-related protein expression. A: the total protein bands; B-E: the expression of OPN, OCN, ALP, and Runx2, respectively, indicated by the statistical analysis of the gray value to make a bar graph according to the protein bands shown; * represents the statistical differences between different treatment groups, *P < 0.05, **P < 0.01, ****P < 0.0001.

In order to study whether the osteogenic related proteins that have been reduced after knocking down miR-145a-3p will be elevated, the experiment was divided into four groups, and after knocking down by using the transfection method, the expression trend of the osteogenic related proteins OPN, OCN, ALP and Runx2 was statistically analyzed by Western blot experiments, Tanon 5200 exposure strips, and the results of the experiments indicated that originally decreased osteogenic protein expression due to dexamethasone stimulation, protein expression was elevated to a certain extent after knocking down the target miRNA (Fig 9), indicating that knocking down miR-145a-3p could improve the decreased expression of protein levels caused by dexamethasone.

### Effect of knockdown of miR-145a-3p on the expression of osteogenesis-related genes

**Fig 10:**
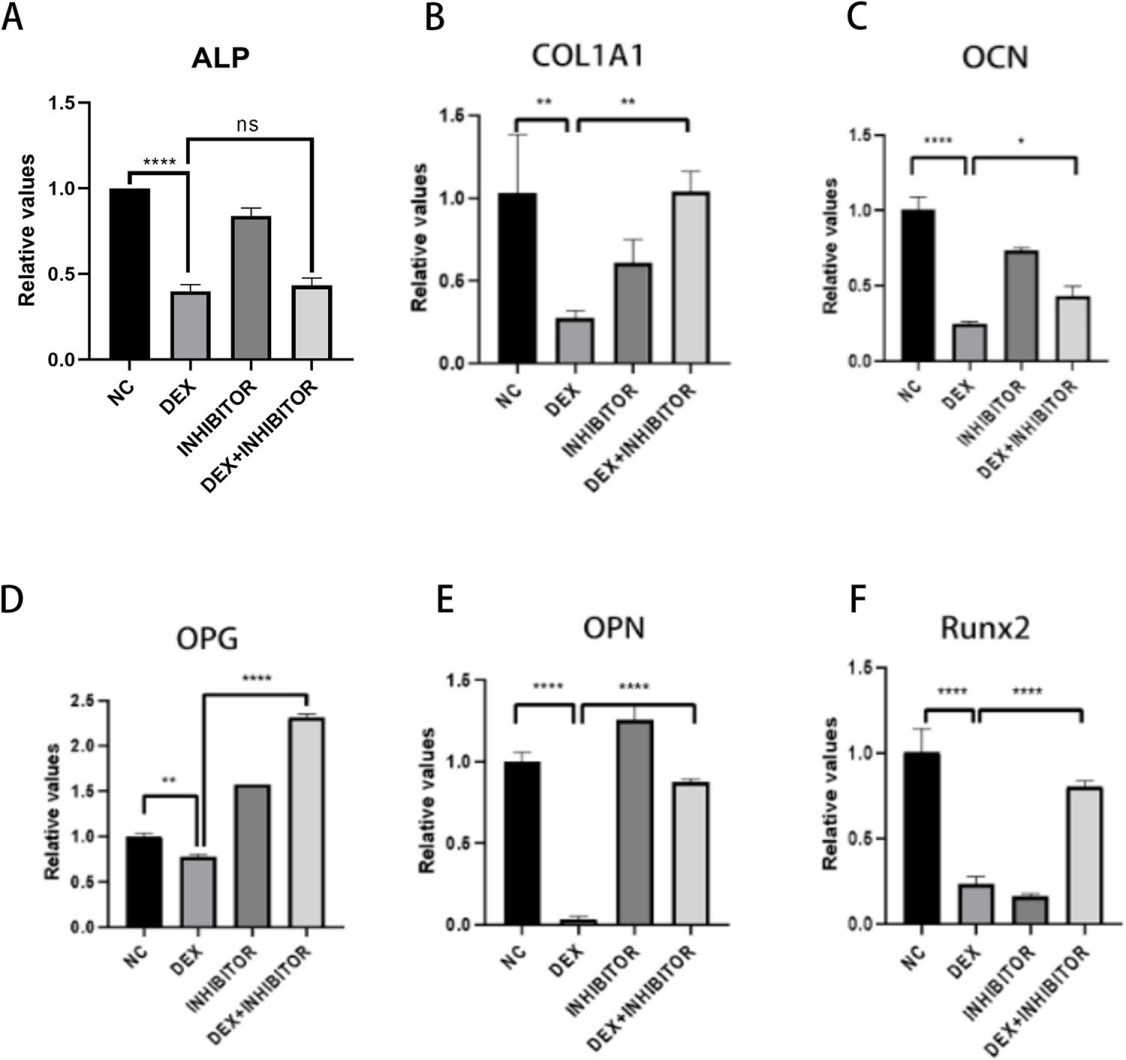
PCR sensing the expression of osteogenesis-related genes. PCR was used to detect ALP (A), COL1A1 (B), OCN (C), OPG (D), OPN (E), and Runx2 (F) gene expression, and bar graph analysis was done according to the CT values; * represents the statistical differences between different treatment groups, *P < 0.05, **P < 0.01, ****P < 0.0001.

In order to investigate whether the osteogenesis-related genes that had been reduced after knocking down miR-145a-3p would be elevated, the experiment was divided into four groups, and the transfection method was used to knock down and then statistically analyze the trend of expression of the osteogenesis-related genes ALP, COL1A1, OCN, OPG, OPN, and Runx2 by qRT-PCR experiments, and the results of the experiments indicated that originally The experimental results indicated that the expression of osteogenic genes, which had been reduced due to dexamethasone stimulation, was elevated to a certain extent after knocking down the target miRNAs, except for ALP, the expression of COL1A1, OCN, OPG, OPN and Runx2 genes was elevated to a certain extent (Fig 10), which indicated that miR-145a-3p could ameliorate the decrease in the expression level of the genes caused by dexamethasone.

### Prediction and validation of target genes of MiR-145a-3p

**Fig 11:**
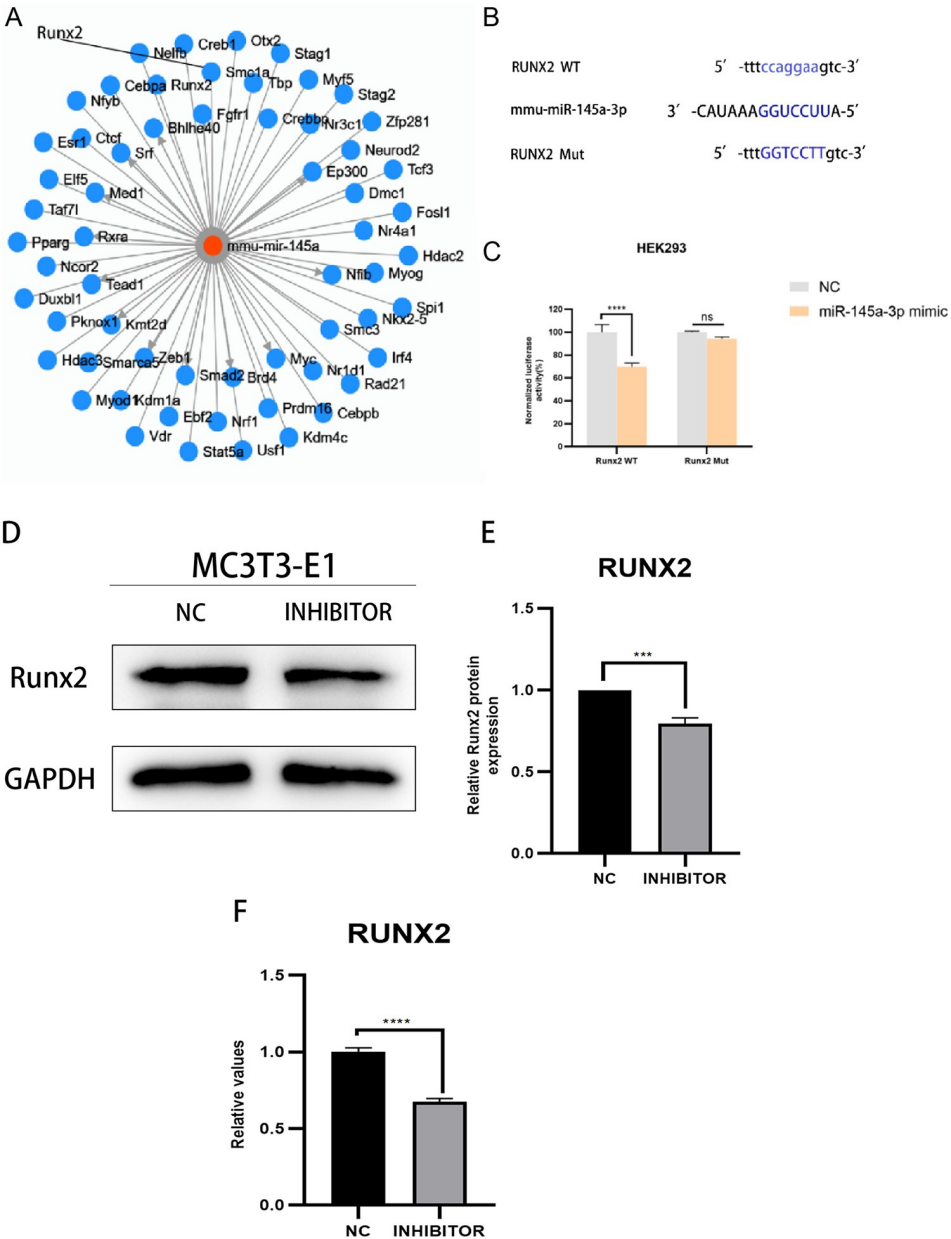
Target gene prediction and validation. A: TransmiR v2.0 predicts the target genes of miR-145a-3p, and one of them, Runx2, is pointed out with a line; B: the gene structure of Runx2 indicates one of the predicted targets of miR-145a-3p in its 3’ non-coding region; C: the dual luciferase reporter gene assay was used to detect the WT/Mut Runx2 3 ’-UTR and miR-145a-3p after co-transfection of HEK293 cells with luciferase activity; D: Detection of protein bands expressed by runx2 under knockdown miR-145a-3p conditions; E: Gray value analysis of protein bands; F: Detection of runx2 gene expression under knockdown miR-145a-3p conditions; * represents the statistical differences of different treatment groups, ***P < 0.001, ****P < 0.0001.

The results of the aforementioned experiments indicated that knockdown of miR-145a-3p improved the decrease in osteogenic capacity caused by dexamethasone, so we were ready to further explore the possible specific mechanism of action of this target miRNA, and a search of TransmiR v2.0 on target gene prediction websites yielded results showing that the target gene of miR-145a-3p might be Runx2 (Fig 11A), thus It was guessed that miR-145a-3p might directly target Runx2 to regulate the changes of osteogenic ability; it was found that miR-145a-3p and Runx2 might have two different binding sites, in order to verify their targeting relationship, wild-type and mutant plasmids were synthesized according to the sites, their sequences were Runx2 WT1 5’- ccaggaa-3’, Runx2 Mut1 5’-GGTCCTT-3’, Runx2 WT2 5’-ccaggaa-3’ and Runx2 Mut2 5 ’-GGTCCTT-3’ (Fig 11B), followed by results from plasmid extraction, cell transfection and luciferase reporter gene experiments (Fig 11C). The results indicated that miR-145a-3p was used to restore dexamethasone-induced decrease in MC3T3-E1 osteogenic capacity by directly targeting Runx2. Finally, the expression level of Runx2 was detected by Western blot assay and qRT-PCR assay respectively after knocking down the target miR-145a-3p. -3p, both protein and gene expression of Runx2 were down-regulated, further demonstrating the direct targeting relationship between miR-145a-3p and Runx2 (Fig 11D–11F).

## Discussion

MiRNAs play an increasingly important role in the regulation of bone metabolism, and the present study is aimed at exploring differentially expressed miRNAs and analyzing their mechanism of action in the context of decreased osteogenic capacity of cells.The specific mechanism of the involvement of MiR-145a-3p in the process of osteogenesis has not yet been reported in the literature. In this experiment, we firstly set up a dexamethasone concentration gradient group (0, 5μM, 10μM, 15μM) to stimulate mouse MC3T3-E1 cells in 96-well plates through the CCK8 experiment and the related literature, and then incubated the cells in a warm box for 0-72h, then added CCK8 reagent and incubated them in a protected place for 2h-4h, and then measured the absorbance at 450nm with an enzyme meter, and found that the change of the absorbance was more significant in the range between 0 and 4h, and the difference between 0 and 4h was more significant. The change of absorbance at 72h is more significant, the difference of absorbance between 0 and 5μM concentration is not obvious, at the same time, there is no statistically significant difference between 10μM and 15μM absorbance, the result of CCK8 experiment can suggest the concentration of the most obvious effect, and the maximum amount of dexamethasone sodium phosphate used by adults in clinic is 20mg per day, and the concentration of dexamethasone diluted in 100ml of dextrose is 500μM, the concentration of dexamethasone taken in the experiment is less than this concentration, and the concentration of dexamethasone is 500μM. In this experiment, the concentration of dexamethasone was less than this concentration to prove that the experimental concentration is reasonable and feasible, so we chose a suitable dexamethasone solution concentration of 10μM, so as to form a mature and stable state of decreased osteogenic ability, and through a variety of experimental methods, such as flow cytometric detection of apoptosis and reactive oxygen species (apoptosis in the dexamethasone solution concentration stimulation group is more than the control group, and reactive oxygen species are also larger than the control group), alkaline phosphatase staining (alkaline phosphatase decreased in the hormone stimulation group), and the hormone stimulation group. Alkaline phosphatase staining (decrease in alkaline phosphatase in the hormone-stimulated group) and alizarin red staining (decrease in mineralized nodules in the hormone-stimulated group), Western Blot (significant decrease in the protein levels of Runx2, ALP, OPN, and OCN in the dexamethasone-stimulated group at a concentration of 10 μM), and qRT-PCR (significant down-regulation of the expression levels of the genes of Runx2, ALP, OPN, OPG, OCN, and COL1A1 in the dexamethasone-stimulated group). expression levels were significantly down-regulated) were verified. Data from several studies suggest that dexamethasone promotes osteoblast apoptosis possibly through the PI3K/AKT signaling pathway or by targeting Caspase-3 [54,55]; reactive oxygen species (ROS) levels are important signals for normal physiological functions of the cell and for cell damage caused by environmental factors, and the detection of intracellular ROS levels is of great importance for understanding the signaling pathways and potential mechanisms of action of some drugs. The production of ROS is considered a normal physiological activity of cellular metabolism; however, studies on the pathomechanism of GC have shown that excessive and prolonged GC treatment leads to excessive accumulation of intracellular ROS through the dysregulation of mitochondrial function, resulting in the up-regulation of oxidative stress [56], which also validates the results of this experiment’s flow cytometric detection of reactive oxygen species, which were increased in dexamethasone-treated MC3T3-E1 cells. There may be a complex interaction between reactive oxygen species and miRNAs during osteogenesis, and the level of reactive oxygen species may affect the expression and activity of miRNAs, thus indirectly affecting osteogenic function. At the same time, miRNAs may also regulate the expression of osteogenesis-related genes to counteract or adapt to the oxidative stress caused by reactive oxygen species. Osteoblast alkaline phosphatase mainly responds to the activity of osteoblasts, which is both physiologically and pathologically elevated, and pathologically elevated alkaline phosphatase is most often seen in patients with osteoporosis because of calcium loss or increased bone resorption, which leads to transient elevation of alkaline phosphatase [66]. patients [57]; calcified nodules generally refer to the necrosis of some tissues in the body caused by some reasons, and then the calcium ions in the body are deposited into the necrotic area, so as to control the lesion and thus achieve a stable state [58]. In fact, it is a kind of defensive response of the organism to the lesion. The results of alizarin red staining showed that the calcified nodules were significantly reduced after the application of GC treatment, but the specific mechanism was unknown; the concentration of DEX in the osteogenic induction medium used in this experiment was only 0.1 μM, which was one percent of the experimental concentration of 10 μM, so that its effect on the staining results could be ignored. Osteoblasts are the main functional cells of bone formation and are important cells involved in the synthesis, secretion and mineralization of the bone matrix.Bone is constantly undergoing reconstruction.Bone remodeling is the process whereby osteoclasts attach to the old bone area and break it down by secreting acids and proteases, resulting in the formation of a pit of bone resorption. Following this, osteoblasts migrate to the resorbed site and secrete large amounts of bone material and mineralize it into new bone [59,60]. Previous studies have found that osteocalcin is significantly elevated in osteoblasts during the bone mineralization phase, followed by a gradual decline, accompanied by an elevation of collagenase, apoptosis, compensatory proliferation and collagen synthesis in osteoblasts.

Comparison of the predicted miRNAs to take the intersection resulted in five miRNAs, and after qRT-PCR experiments, only the target miR-145a-3p was found to have a stable and uniformly high expression trend in the state of decreased osteogenic capacity. miRNAs are non-coding RNAs that play corresponding functional roles by inhibiting the expression of potential downstream target genes [61]. A study showed that miRNAs are key regulators, both positively and negatively, in the formation, survival, and death of osteoclasts and osteoblasts [62]. Some miRNAs, such as miR-7b-5p and miR-19a-3p, can delay the development of osteoporosis [63,64]. Therefore, these miRNAs can be used as new targets for the prevention and treatment of osteoporosis. miR-145 is a newly discovered miRNA molecule in recent years, which plays an important role in the processes of cell invasion, proliferation, apoptosis, and differentiation [65,66], and one of them, miR-145a-3p, has also been found to be associated with osteoblasts [67,68]; thus, after figuring out the appropriate After figuring out the appropriate concentration ratio of transfection reagents, miR-145a-3p was further knocked down in the state of decreased osteogenic ability, and several different groupings (alkaline phosphatase staining and flow experiments were divided into the control group, hormone-stimulated group, and hormone-stimulated and simultaneous miR-145a-3p Inhibitor transfection group; Western blot experiments and qRT-PCR experiments were divided into the control group, hormone-stimulated group, and hormone-stimulated only group). Western blot and qRT-PCR experiments were divided into control group, hormone-stimulated group, miR-145a-3p Inhibitor transfected group and hormone-stimulated simultaneous miR-145a-3p Inhibitor transfected group) to discuss the changes in osteogenesis, and it was found that the osteogenesis, which was originally reduced due to the hormone stimulation, could be significantly restored to a level close to the normal level after knocking down the miR-145a-3p, and alkaline phosphatase staining level could be significantly increased compared with the hormone-stimulated group. The level of alkaline phosphatase staining increased significantly from the hormone-stimulated group to a level close to that of the blank control group, and the expression levels of the osteogenesis-related proteins OPN, Runx2, ALP, and OCN increased to a certain extent, and the expression levels of the osteogenesis-related genes Runx2, OPG, COL1A1, OPN, and OCN were similarly reversed except for the expression of ALP, which did not retrace after knocking down, which may be due to the fact that no osteogenesis was added to cultured cells. This may be due to the fact that osteogenic induction medium was not added when the cells were cultured, and it can be initially concluded that miR-145a-3p can be used as a target to ameliorate the decline in osteogenic capacity. However, flow cytometry results showed that apoptosis and reactive oxygen species levels did not decrease after knocking down miR-145a-3p, and there was no statistical significance among the experimental subgroups, which can indicate that the mechanism of miR-145a-3p’s action is not through affecting the expression level of osteogenic cells. was not by affecting apoptosis and reactive oxygen species in osteoblasts.

To further study the specific target gene and mechanism of miR-145a-3p, and whether it affects the whole process through this target gene, after consulting a large number of related literature and miRNA target gene prediction websites, we can know that miR-145a-3p may work by directly targeting Runx2, and after synthesizing the related plasmids, we verified that there is indeed a targeting effect between miR-145a-3p and Runx2 through dual-luciferase reporter gene assay and qRT-PCR assay. After synthesizing the related plasmid, it was verified by dual luciferase reporter gene assay that miR-145a-3p and Runx2 did have a targeting effect, and then it was verified by Western blot assay and qRT-PCR assay that miR-145a-3p affected the alteration of osteogenic function by targeting Runx2. runx2 gene is a transcription factor expressed during embryonic development, and its main function is to regulate the differentiation and maturation of bone cells. maturation.The expression of Runx2 gene is mainly regulated by a variety of signaling pathways, such as Wnt signaling pathway, BMP signaling pathway, etc [69]. The activation of these signaling pathways can promote the transcription and translation of Runx2 gene, thus enhancing the differentiation of skeletal cells.Runx2 gene regulates the expression of a series of target genes, including alkaline phosphatase (ALP), osteocalcin, etc., which are closely related to the differentiation and maturation of skeletal cells, by binding to specific sequences on the DNA [70]. In addition to its role in skeletal cell development, Runx2 gene also plays an important regulatory role in bone metabolism. It has been found that the expression level of Runx2 gene is associated with skeletal diseases such as osteoporosis and fracture healing [71]. In patients with osteoporosis, the expression level of Runx2 gene is usually low, leading to insufficient differentiation and maturation of skeletal cells, which results in bone loss. In contrast, during fracture healing, the expression level of Runx2 gene increases significantly, which promotes the proliferation and repair of skeletal cells and accelerates fracture healing. It can be concluded that miR-145a-3p regulates dexamethasone by directly targeting Runx2 leading to decreased osteogenic function. However, this target miRNA was found to be highly conserved in mice, and its degree of conservation in humans has not yet been confirmed, so further in vitro and ex vivo experiments and clinical practice are still needed to explore and validate the potential role of the miR-145a-3p/Runx2 pathway in the process of osteogenic function alteration in osteoporosis.

## Conclusion

The results indicated that dexamethasone impaired the osteogenic differentiation ability of MC3T3-E1 cells by inducing the up-regulation of miR-145a-3p expression. miR-145a-3p inhibited the osteogenic differentiation ability of MC3T3-E1 cells by targeting and inhibiting the expression level of Runx2 protein. Altering intracellular miR-145a-3p levels significantly affected the osteogenic differentiation ability of MC3T3-E1 cells. Therefore, new ideas to improve the treatment of decreased osteogenic capacity in GIOP could be developed based on this differential candidate miR-145a-3p.

## Supporting information

S1 Raw images (PDF)
