# Supplementary figures and images for: Mechanism of MiR-145a-3p/Runx2 pathway in dexamethasone impairment of MC3T3-E1 osteogenic capacity in mice
Source: PLoS One. 2024 Nov 19;19(11):e0309951. doi: 10.1371/journal.pone.0309951 (PMC11575826; doi:10.1371/journal.pone.0309951)

Figure 4

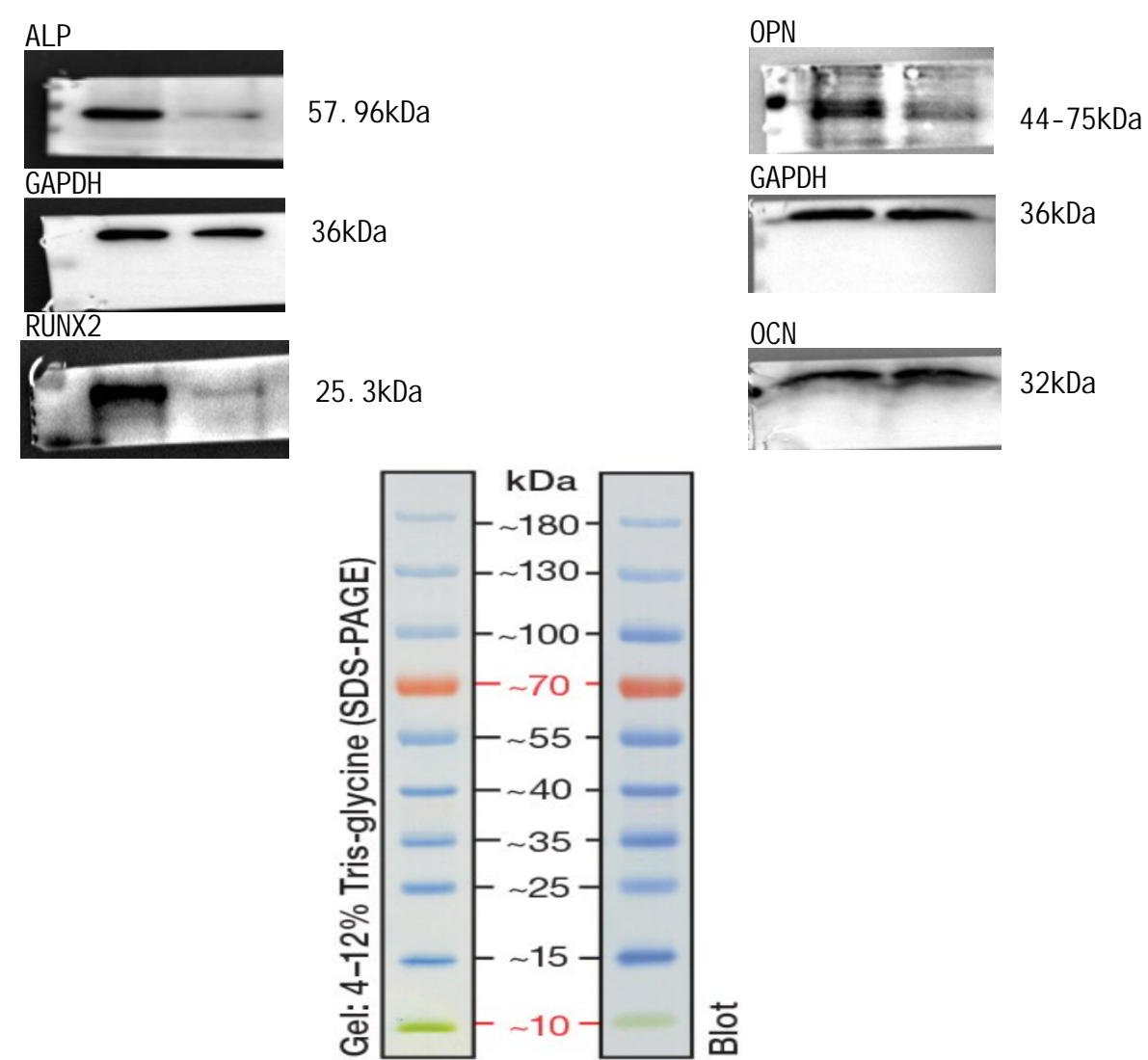

Figure 9

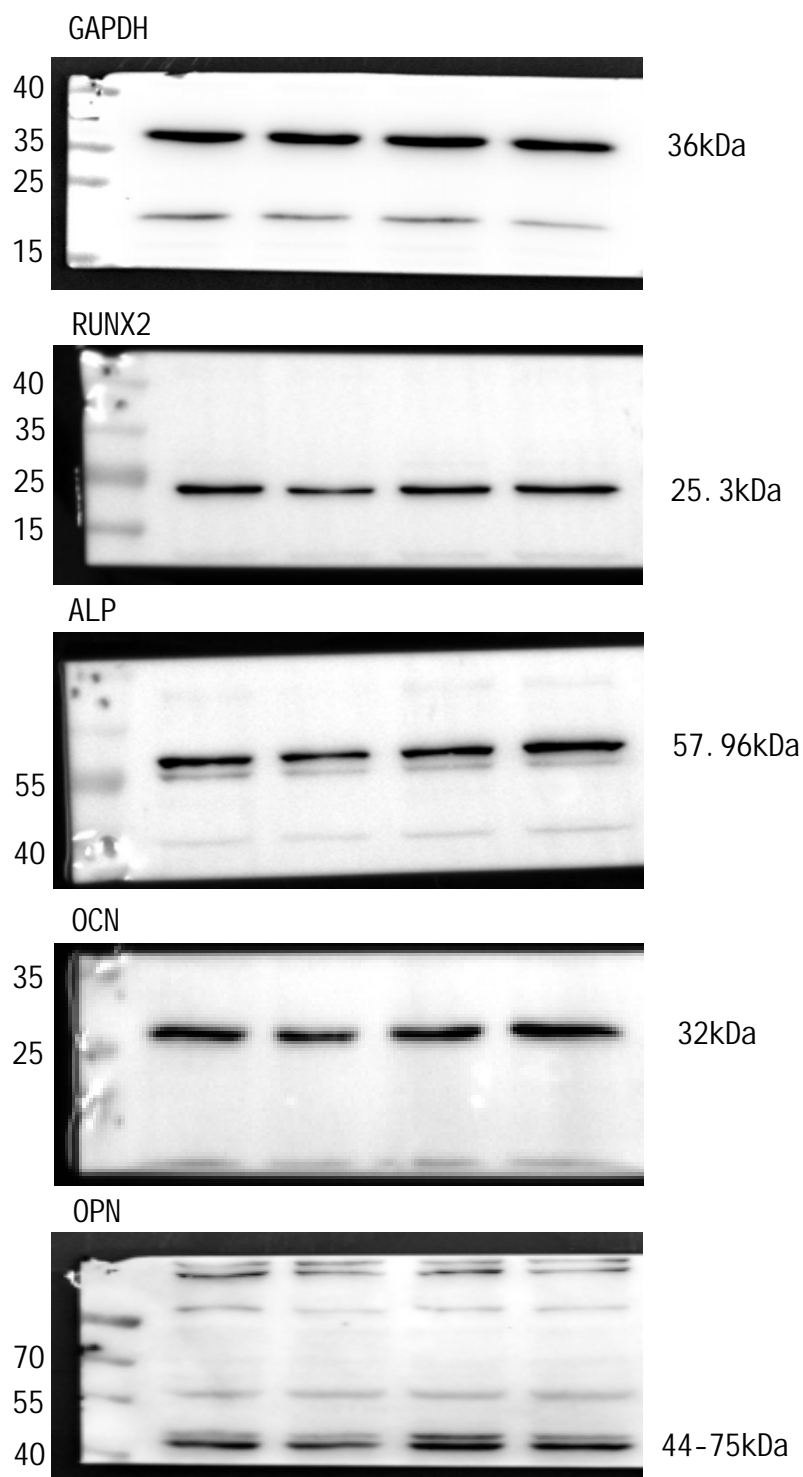

Supplement: S1 Raw images — (PDF) [file pone.0309951.s001.pdf]
